# The postantibiotic effect-like effect of photothermal agents: evidence from IR780 nanoparticles and indocyanine green

**DOI:** 10.3389/fphar.2026.1939215

**Published:** 2026-09-30

**Authors:** Tiantian Xu, Zhihao Wang, Xiaolan Li

**Affiliations:** 1 Laboratory Animal Research Center, Jiangsu University, Zhenjiang, Jiangsu, China; 2 School of Pharmacy, Jiangsu University, Zhenjiang, Jiangsu, China

**Keywords:** CRE, IR780 nanoparticles, MRSA, photothermal therapy, postantibiotic effect-like effect

## Abstract

**Introduction:**

Postantibiotic effect (PAE) refers to the temporary suppression of bacterial growth after transient antimicrobial treatment. In recent years, due to the spread of antibiotic resistance, photothermal agents (PTAs) have been broadly studied for infection treatments. However, the PAE-like effect of PTAs has not been investigated.

**Methods:**

This study focuses on the PAE-like effect of representative PTAs, namely IR780 nanoparticles (NPs) and indocyanine green (ICG), against MRSA and a CRE isolate. The effects of PTA type, bacterial species, PTA concentration, and near-infrared irradiation duration on the PAE-like effect were systematically evaluated.

**Results:**

Results showed that the PAE-like effect of these PTAs against MRSA and a CRE isolate was evident. Further experiments showed that the PAE-like effect was affected by PTA type, bacterial species, PTA concentration, and near-infrared irradiation duration. Furthermore, the PAE-like effect of IR780 NPs was enhanced by 34.6% when a targeting group was conjugated to its nanostructure.

**Discussion:**

The mechanism of the PAE-like effect of PTAs against MRSA is suggested to be related to thermally induced damage. Overall, this work provides the first evidence of a PAE-like effect in PTAs against MRSA, which may provide a preliminary basis for future optimization of photothermal therapy regimens against resistant infections.

## 1 Introduction

The emergence and spread of antimicrobial resistance in pathogens are posing major threats to global public health. If the resistance problem is left unsolved, up to ten million people could die annually by 2050 as a result of antimicrobial resistance (Ziesmer et al., 2023). There are currently two ways to solve this problem: reducing antibiotic usage and developing new antimicrobial agents. However, the pipeline of new antibiotics is dry. Since 1 July 2017, only 13 new antibiotics have been approved for global marketing, with merely two belonging to novel chemical classes and qualifying as truly innovative drugs (WHO, 2025). Therefore, there is an urgent need to develop alternative antimicrobial strategies beyond traditional antibiotics.

Photothermal therapy (PTT) has emerged as a promising alternative for infection treatment, utilizing photothermal agents (PTAs) to convert near-infrared (NIR) light into localized heat that kills pathogens while sparing normal cells. PTAs are broadly categorized into the following classes: noble metal, carbon-based nanomaterials, metal sulfides/oxides, polymer nanomaterials, and small molecules (Yuan et al., 2024). Each has distinct characteristics and limitations. Noble metal nanoparticles (e.g., Au, Ag) offer high photothermal conversion efficiency but are non-biodegradable, and raise significant safety concerns due to their persistence in the body (Askari and Eskandari, 2025). Carbon-based nanomaterials (e.g., graphene, carbon nanotubes) exhibit good biocompatibility and high loading capacity, yet their long-term toxicity and poor biodegradability remain unresolved (Hu et al., 2015). Metal sulfides/oxides such as CuS are readily synthesized but are not easily degraded and have been associated with hepatotoxicity (Cui et al., 2025). Organic small-molecule dyes, including indocyanine green (ICG) and IR780, are generally more biosafe—ICG is FDA-approved for clinical diagnostics and IR780 has been extensively applied in antibacterial studies (Liu et al., 2019; Yu et al., 2022). However, these agents are not without toxicity concerns. The localized hyperthermia generated by ICG and IR780 (and most PTAs) during PTT, while effective for bacterial eradication, is inherently non-selective and poses a substantial risk of irreversible thermal damage to adjacent healthy tissues (Wilson and Weersink, 2020). Although conjugating bacterial-targeting moieties to PTAs can partially mitigate this off-target damage by enhancing local accumulation at infection sites, such strategies cannot eliminate the risk of thermal injury, particularly in scenarios involving incomplete targeting or high local temperatures (Wang et al., 2025). Therefore, minimizing treatment frequency and reducing therapeutic dosages remain essential approaches to improve the safety profile of PTT, ensuring effective bacterial clearance while limiting collateral harm to healthy tissues.

The postantibiotic effect (PAE) is defined as the period encompassing suppression of bacterial growth following limited exposure to antimicrobial drugs. This phenomenon has significant clinical implications, as antibiotics with longer PAE may permit longer dosing intervals. The PAE was first observed in the 1940s in studies of penicillin against *Pneumococcus* and *Streptococcus* (Watson et al., 1993). Since then, it has been found across diverse antibiotic classes—including aminoglycosides, β-lactams, and fluoroquinolones—against a wide spectrum of bacterial species. Higher aminoglycoside peak concentration was found to be related to longer PAE (MacKenzie and Gould, 1993), while Bundtzen et al. found that the PAE of β-lactams was related to both the concentration of antimicrobials and the duration of exposure (Davoodi et al., 2020). The mechanism of PAE has not been clearly delineated. For a long period of time, the mechanism is suspected to be related to drug-induced nonlethal damage and persistence of antimicrobials at the bacterial binding site (MacKenzie and Gould, 1993). In 2017, Srimani et al. came up with a theory that PAE can be explained by the temporal dynamics of drug detoxification in individual cells after an antibiotic is removed from the extracellular environment (Srimani et al., 2017). Despite the unresolved mechanisms, PAE is of great significance in determining and optimizing the dosing intervals in a multidose antimicrobial regimen.

However, whether PTT induces a PAE-like effect remains entirely unexplored. This study, therefore, aimed to bridge this knowledge gap by measuring the PAE-like effect of two representative PTAs--IR780 (in both non-targeted and targeted formulations) and ICG--against Gram-positive and Gram-negative bacteria. We further investigated the influence of key treatment parameters, including irradiation time and PTA concentration, on the PAE-like effect duration. Our results confirmed that all tested PTAs have a significant PAE-like effect, with targeted IR780 nanoparticles exhibiting the most pronounced effect. To the best of our knowledge, this article is the first work evaluating PAE-like effect of PTAs, providing insight that could inform the design of more rational PTT dosing regimens.

## 2 Materials and methods

### 2.1 Materials

Octylamine, IR780, and concanavalin A (ConA) were purchased from Sigma-Aldrich (St. Louis, United States). N-octyl-D-gluconamide was synthesized by a previous method (Xu et al., 2023). Polyethylene glycol 4000 (PEG 4000) and octyl glucopyranoside were obtained from J&K Scientific Ltd. (Beijing, China). Antifade Mounting Medium, NucGreen, defibrinated sheep blood, and bacterial culture reagents were purchased from Beyotime Biotechnology (Beijing, China). All the other chemicals were of analytical grade and were used as received. Ultrapure water was used throughout the experiments.

### 2.2 Fabrication of IR780-N-octyl-D-gluconamide nanoparticles (IR780 NPs)

IR780 NPs with *Staphylococcus aureus*-targeting ability were prepared by our previous method (Xu et al., 2023). Briefly, 1 mg of IR780 and 2 mg of N-octyl-D-gluconamide were dissolved in 1 mL of dimethyl sulfoxide. The mixture was sonicated for 10 min at 100 W ultrasound power (GT SONIC, Guangdong, China). PEG 4000 (20 mg) was then added to the solution, and the mixture was heated at 70 °C for 5 min. Then, an excess amount of ultrapure water (5 mL) was added to the solution. The mixture was sonicated at room temperature (25 ± 3 °C) for 2 h at 100 W. The obtained nanoparticles were then purified using a dialysis bag (MWCO 300 kDa) against an excess amount of ultrapure water overnight. A dialysis membrane with a molecular weight cutoff (MWCO) of 300 kDa was selected because its pore size is significantly smaller than the hydrodynamic diameter of the IR780 nanoparticles (∼30–55 nm, as characterized in our previous study; Xu et al., 2023), ensuring that the nanoparticles were retained within the dialysis bag. In contrast, free small-molecule components (unencapsulated IR780, GA, octyl glucopyranoside, and PEG 4000, all with molecular weights < 5 kDa) could freely diffuse through the membrane. The use of an excess volume of ultrapure water (overnight dialysis with multiple water changes) ensured efficient removal of these low-molecular-weight impurities. This purification protocol has been validated in our previous study (Xu et al., 2023), where the successful removal of free components was confirmed by UV–vis absorption spectroscopy and dynamic light scattering. IR780 NP concentrations are expressed based on IR780 content, which was determined by fluorescence intensity using a pre-established standard curve (Xu et al., 2023).

IR780 NPs without bacterium-targeting ability (IR780-U NPs) were prepared in the same way except for replacing N-octyl-D-gluconamide (GA) with the same amount of octyl glucopyranoside. The morphology and structure of IR780 nanoparticles, as well as their targeting ability towards bacteria, have been characterized in a previous article of our team (Xu et al., 2023).

### 2.3 Determination of photothermal performance

The photothermal performance of ICG and IR780 NPs in aqueous solution was tested by exposing the solutions (25 μg/mL) to NIR laser (808 nm, 0.8 W/cm^2^) for 10 min. An infrared thermometer was used to measure the temperature at specified time points (0, 1, 2, 5, and 10 min). Distilled water was used as the blank.

For the photothermal performance measurements, each sample (200 μL) was placed in a 96-well plate and irradiated from the top using an 808 nm continuous-wave diode laser. The beam spot diameter was approximately 1.5 cm, and the distance from the laser exit aperture to the sample surface was fixed at 10 cm. No mixing was applied during irradiation.

Unless otherwise noted, NIR irradiation at 808 nm and 0.8 W/cm^2^ was used throughout this study.

### 2.4 Bacterial culture

Since most of the PTT researches aim at dealing with antibiotic-resistant infections, in this article standard strain (ATCC43300) of methicillin-resistant *S. aureus* (MRSA) and a clinical isolate of carbapenem-resistant *Escherichia coli* (CRE, strain GDB8P65M) was used. The CRE isolate was obtained from a porcine fecal sample collected from a swine farm in Guangdong Province, China, and was previously characterized by our laboratory (Ma et al., 2021). The strain was identified as *E. coli* by MALDI-TOF-MS, and the presence of the *bla*_NDM-5_ gene was confirmed by PCR and Sanger sequencing. Antimicrobial susceptibility testing showed that GDB8P65M is resistant to multiple antibiotics, including meropenem (MIC = 16 μg/mL) (Ma et al., 2021). The strain was obtained from our laboratory’s archived collection of previously characterized bacterial isolates, and its use in this *in vitro* study did not require additional approval.

We created stock solutions using 40% glycerol and stored them at −25 °C. For the experiment, we inoculated the stock solution into the Luria-Bertani agar and incubated it at 37 °C overnight. Then we picked one isolated colony from the cultured agar plate and transferred it into a sterilized tube containing LB broth medium. The tube was then shaken at 180 rpm at 37 °C to the bacterial exponential phase. For colony-forming units (CFUs) counting, 50 μL of bacterial solution was serially diluted 10-fold in sterile PBS. Then 50 μL of each diluted sample was plated onto LB agar plates using the spread-plate method with sterile glass spreaders. For each dilution, two technical duplicate plates were prepared. After incubation at 37 °C for 12 h, plates containing 30–300 colonies were counted; counts outside this range were discarded. Bacterial concentration (CFU/mL) was calculated as shown in Equation 2.1:

$$Bacterial\;concentration=\frac{N\times D}{V}$$ (2.1)

where *N* is the number of colonies, *D* is the dilution factor, and *V* is the plated volume (mL). All CFU counts were performed in three independent biological replicates (n = 3) conducted on separate days using freshly prepared bacterial cultures.

### 2.5 Determination of the PAE-like effect

The PAE-like effect of IR780 NPs and ICG was tested according to a previous method (Huband et al., 2015). Firstly, bacteria were cultured to the exponential phase and then diluted to 2 × 10^7^ CFU/mL. PTA solution (IR780 NPs or ICG) of certain concentrations (0, 25, 50, 100 μg/mL, solvent was LB broth) was mixed with the bacterial solution in a 1:1 volume ratio, yielding final PTA concentration of 0, 12.5, 25, and 50 μg/mL. Then NIR irradiation was applied to the mixtures for a certain period (0, 1, 2, 5 min, respectively). After that, to remove PTA, MH broth was used to dilute the mixtures by 1000-fold. Then PE tubes containing the mixtures were shaken at 180 rpm at 37 °C for 4 h. CFUs were counted every 1 h using the method described in 2.4. The formula for calculating PAE is shown in Equation 2.2:

PAE=T−C (2.2)

where *T* is the time necessary for the bacterial population to grow by 1 log(CFU/mL), and *C* is the corresponding time for the blank control group. Linear interpolation was applied to estimate T and C when the 1-log increase occurred between two consecutive measurement time points. The blank control for PAE calculation was defined as the group with 0 μg/mL PTA and 0 min NIR irradiation, which was assigned a PAE value of 0 h by definition.

### 2.6 Membrane permeability assay

Propidium iodide (PI) was used to test bacterial cellular membrane permeability (Hoenes et al., 2021). Firstly, 10^8^ CFU/mL of washed bacteria were resuspended in 5 mM HEPES with 0, 25, 45, 90, 180 μg/mL of IR780 NP in them. Ten minutes of NIR irradiation was applied to the mixtures, followed by 5000 g × 5 min centrifugation. The washed bacteria were then incubated with 10 nM PI at 37 °C for 20 min. A microplate reader was used to measure the fluorescence intensity of PI; the excitation/emission wavelength was set to be 535 nm/615 nm. The control group was not treated with NIR irradiation, with other conditions being the same.

### 2.7 Intracellular oxidative activity assay

#### 2.7.1 Quantitative testing

A 2′,7′-Dichlorodihydrofluorescein diacetate (DCFH-DA) based assay was used to assess intracellular oxidative activity (Wu et al., 2022). Firstly, 10^8^ CFU/mL of washed bacteria were resuspended in 0.01 M PBS (pH = 7.4). Then 10 μM of 2′,7′-dichlorodihydrofluorescein diacetate (DCFH-DA), which is a reactive oxygen species (ROS) probe, was incubated with the suspension for 20 min 5000 g × 5 min of centrifugation in PBS was used to wash the bacteria 3 times. Then, 0, 25, 45, 90, 180 μg/mL of IR780 NPs were added, and 10 min of NIR irradiation was applied. A microplate reader was used to measure the fluorescence intensity of DCFH-DA. The excitation/emission wavelength was set to be 488 nm/525 nm. The control group was not treated with NIR irradiation, with other conditions being the same.

#### 2.7.2 Confocal laser scanning microscope characterization

Firstly, 10^8^ CFU/mL of washed bacteria were resuspended in 0.01 M PBS (pH = 7.4). Then 10 μM of DCFH-DA, which is a probe for oxidative activity, was incubated with the suspension for 20 min 5000 g × 5 min of centrifugation in PBS was used to wash the bacteria 3 times. Then, 0, 25, 45, 90 μg/mL of IR780 NPs were added, and 10 min of NIR irradiation was applied. Confocal laser scanning microscopy (CLSM) was used to characterize the bacteria. The excitation wavelength was set to be 488 nm. The emission signal detecting wavelength range was set to be 495–620 nm. Photomultiplier tube (PMT) was used as the receiver. The gain value was set to be 800.0 V. The scanning speed was set to 400 lines/min.

### 2.8 Membrane potential assay

3,3′-Dipropylthiadicarbocyanine iodide (DiSC_3_(5)) was used to test membrane potential (Seydlova et al., 2019). Firstly, 10^8^ CFU/mL of washed bacteria were resuspended in 5 mM HEPES with 0, 25, 45, 90, 185 μg/mL of IR780 NP in them. Ten minutes of NIR irradiation was applied to the mixtures, followed by 5000 g × 5 min centrifugation. The washed bacteria were then incubated with 1 mM DiSC_3_(5) at 37 °C for 20 min. A microplate reader was used to measure the fluorescence intensity of DiSC_3_(5). The excitation/emission wavelength was set to be 622 nm/670 nm. The control group was not treated with NIR irradiation, with other conditions being the same.

### 2.9 Statistical analysis

All data were reported as mean ± standard deviation from three independent biological replicates (n = 3), with each biological replicate conducted on separate days using freshly prepared bacterial cultures. Statistical analyses of data were performed using the non-parametric test function in SPSS (Kruskal-Wallis One-Way Anova), followed by Dunn’s *post hoc* test for pairwise multiple comparisons. Bonferroni correction was applied for multiple comparisons. Differences of p < 0.05 were considered statistically significant.

## 3 Results

### 3.1 Photothermal effects of the PTAs

This work aimed to investigate the potential PAE of PTAs, specifically IR780-based NPs and ICG and to identify factors influencing their duration.

We first tested the photothermal performance of these PTAs. Results are shown in Figure 1. The temperature of PTA solutions increased gradually during the initial 5 min of NIR irradiation and remained roughly unchanged over the next 5 minutes. After 5 min of irradiation, the average temperature of the IR780 NP solution reached 41.5 °C, which was 4.8 °C higher than that of the ICG solution, indicating that IR780 NPs exhibited a greater temperature increase than ICG under identical irradiation conditions. In contrast, the temperature of pure water (blank control) increased by less than 3 °C throughout the 10-min NIR irradiation period, proving that the temperature increase in the PTA solutions was due to the combined effect of PTA and NIR irradiation.

**FIGURE 1:**
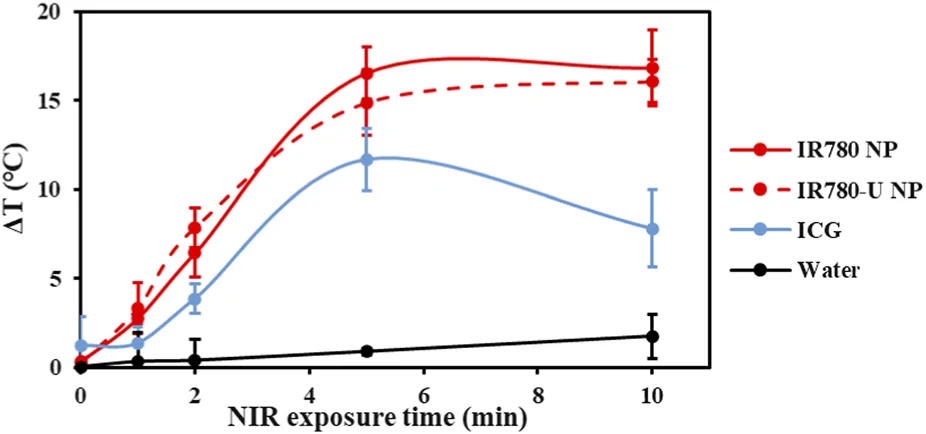
Temperature increase in response to NIR irradiation. The ambient and initial temperature was 25 °C (n = 3).

### 3.2 Antibacterial activities of the PTAs

The antibacterial efficacy of the three PTAs against MRSA and the CRE isolate following 5 min of NIR irradiation is shown in Figure 2. Control experiments confirmed that NIR irradiation alone did not result in any significant decrease in CFU concentration on either strain, which means NIR irradiation was nearly harmless to bacteria under experimental conditions. Similarly, incubating MRSA or the CRE isolate with any of the three PTAs (50 μg/mL) in dark conditions did not result in a significant decrease in bacterial CFU concentration, which means the antibacterial activity requires the combined presence of both the PTA and NIR irradiation.

**FIGURE 2:**
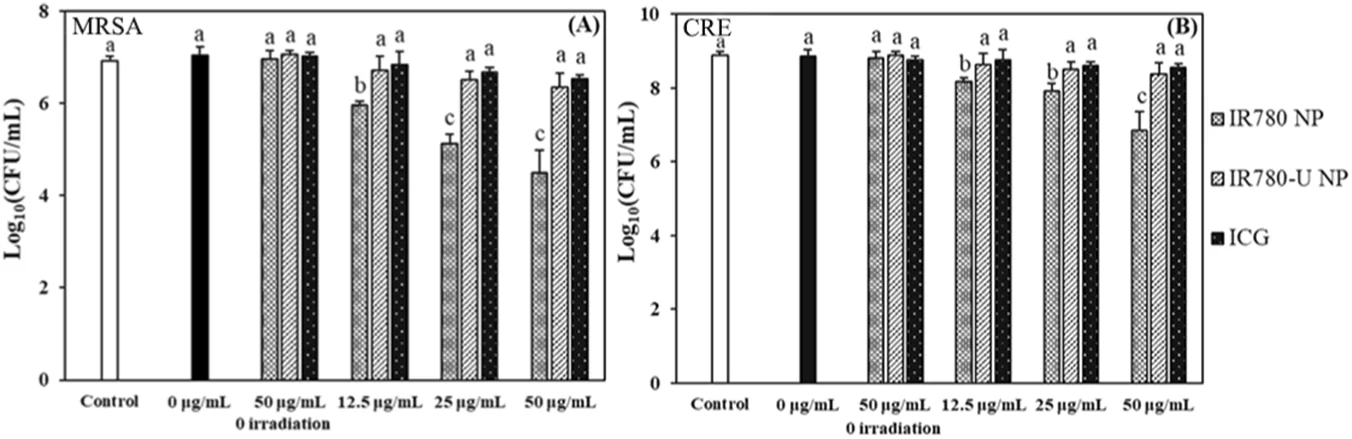
The antibacterial activities of IR780 NPs, IR780-U NPs, and ICG against **(A)** MRSA and **(B)** the CRE isolate. NIR treatment duration was 5 min unless otherwise noted (n = 3). Different letters (a, b, c) above the bars indicate statistically significant differences between groups (p < 0.05, Kruskal-Wallis one-way ANOVA followed by Dunn’s *post hoc* test). Groups sharing the same letter are not significantly different.

As shown in Figure 2A, a clear dose-dependent antibacterial effect was observed against MRSA. Treatment with IR780 NPs at concentrations of 12.5, 25, and 50 μg/mL, followed by 5 min of NIR irradiation, resulted in reductions of the CFU concentration of MRSA of 1.1, 1.9, and 2.5 log CFU/mL, respectively. The IR780-U NPs and ICG also exhibited concentration-dependent activity, albeit significantly weaker, with maximum CFU concentration reductions to be 0.7 and 0.5 log CFU/mL at 50 μg/mL, respectively. This disparity in antibacterial efficacy is probably due to the difference in targeting capability and photothermal heating capability (Li B. et al., 2024).

A similar trend was observed for the CRE isolate (Figure 2B). Antibacterial activity again depended on the synergistic effect of PTA and NIR light. However, the antibacterial activity was less prominent compared with the MRSA group. For example, the CFU concentration reduction achieved by 50 μg/mL of IR780 NPs against the CRE isolate was 2.0 log CFU/mL, lower than the 2.5 log CFU/mL reduction seen in MRSA, highlighting a bacterial species-dependent susceptibility to photothermal treatment.

### 3.3 PAE-like effect of the PTAs

All PTAs in our work showed PAE-like effect towards *S. aureus* and *E. coli* under NIR irradiation (Table 1), which means they all could suppress bacterial growth after their complete removal. PAE value ranged from 0 to 1.8 h depending on PTA type, PTA concentration, NIR irradiation time, and bacterial species (The complete regrowth curves are provided in Supplementary Figure S1).

**TABLE 1:** PAE values (h) of different photothermal agents (PTAs) against MRSA and the CRE isolate under varying irradiation durations and PTA concentrations.

Bacterial species	PTA	Irradiation time (min) at PTA concentration of 25 μg/mL				PTA concentration (μg/mL) at an irradiation time of 5 min			
		PTA only	1	2	5	NIR only	12.5	25	50
MRSA	IR780 NP	−0.03 ± 0.01^a^	0 ± 0.02^a^	0.06 ± 0.04^b^	0.35 ± 0.05^c^	−0.02 ± 0.08^a^	0.1 ± 0.03^a^	0.34 ± 0.03^b^	1.75 ± 0.08^c^
	IR780-U NP	−0.02 ± 0.02^a^	0 ± 0.05^a,b^	0.07 ± 0.01^b^	0.27 ± 0.02^d^	−0.03 ± 0.03^a^	0.07 ± 0.05^a^	0.28 ± 0.03^b,e^	1.3 ± 0.05^d^
	ICG	−0.02 ± 0.05^a,b^	0 ± 0.03^a^	0.03 ± 0.04^b,c^	0.21 ± 0.01^e^	−0.02 ± 0.02^a^	0 ± 0.06^a^	0.02 ± 0.05^a^	0.2 ± 0.03^e^
The CRE isolate	IR780 NP	0.27 ± 0.06^a,b,d^	0.24 ± 0.01^a^	0.28 ± 0.02^b^	0.51 ± 0.06^c,e^	0.26 ± 0.03^a^	0.39 ± 0.01^b^	0.50 ± 0.01^c^	0.69 ± 0.03^d^
	IR780-U NP	0.31 ± 0.02^b,d^	0.28 ± 0.03^b^	0.31 ± 0.01^b,d^	0.53 ± 0.03^c^	0.26 ± 0.05^a^	0.28 ± 0.02^a^	0.53 ± 0.03^c^	0.64 ± 0.02^e^
	ICG	0.33 ± 0.01^d^	0.44 ± 0.04^e,f^	0.39 ± 0.02^f^	0.56 ± 0.02^c^	0.26 ± 0.04^a^	0.26 ± 0.01^a^	0.56 ± 0.04^c^	0.5 ± 0.05^c^

PTAs, were removed by a 1000-fold dilution into MH, broth (n = 3). “The blank control (0 μg/mL PTA + 0 min NIR) was set to 0 h by definition. Within each row, values with different superscript letters (a-e) are significantly different from each other (p < 0.05, Kruskal-Wallis one-way ANOVA followed by Dunn’s post-hoc test). Values sharing the same letter are not significantly different.

The differences between PAE values within 2 min of NIR irradiation were generally insignificant. For example, after 2 min of NIR irradiation, the PAE of all PTAs towards MRSA was lower than 0.1 h (p ≥ 0.58). A similar trend was observed for the CRE isolate, where the PAE value after 2 min of NIR irradiation rose by less than 0.06 h (p ≥ 0.39) compared to the non-irradiation control. In contrast, extending irradiation time to 5 min resulted in significant increases in PAE (p ≤ 0.03). Under these conditions the PAE values for MRSA were 0.4, 0.3, and 0.2 h for the IR780 NP group, the IR780-U NP group, and the ICG group, respectively, indicating the necessity of sufficient light exposure for PAE induction.

When NIR irradiation time was the same, increasing PTA concentration from 25 to 50 μg/mL led to a substantial enhancement of the PAE-like effect against MRSA. The PAE values increased by 1.4 h, 1.0 h and 0.2 h for IR780 NP, IR780-U NP, and ICG, respectively. When the concentration of PTAs was 50 μg/mL and NIR irradiation time was 5 min, the PAE value of the IR780-U NP group was 1.1 h higher than the ICG group against MRSA, indicating that the PAE of IR780 was stronger than ICG. The reason behind this might be that the photothermal effect of IR780-U NP was stronger than ICG: the platform temperature discrepancy between them was 4 °C (Figure 1). Notably, under the same conditions, targeted IR780 NPs exhibited a PAE that was 34.6% higher than that of non-targeted IR780-U NPs (p = 0.032), indicating the PAE effect of the targeted PTA was stronger. This might be due to IR780 NPs with affinity to the cell membrane being hard to remove by diluting, or it might be due to the temperature at the bacterial surface being higher with IR780 NPs adsorbing on it.

Bacterial type was another factor influencing the PAE-like effect. As can be seen in Table 1, when the concentration of IR780 NP was 50 μg/mL and the NIR irradiation time was 5 min, the PAE-like effect towards MRSA was 1.8 h, which was 157% higher than towards the CRE isolate.

We further defined a parameter, “total PTA exposure”, as the product of the duration of NIR irradiation and the concentration of PTA (Figure 3). Our results indicated that the value of PAE-like effect of IR780 NPs against *MRSA* showed a positive correlation with total PTA exposure, with a trend roughly consistent with a nonlinear rise. As for the CRE isolate, the value of PAE-like effect did not change much (less than 0.5) in the range of 0–250 (μg·min)/mL, which denoted discrepancies existed between bacterial types.

**FIGURE 3:**
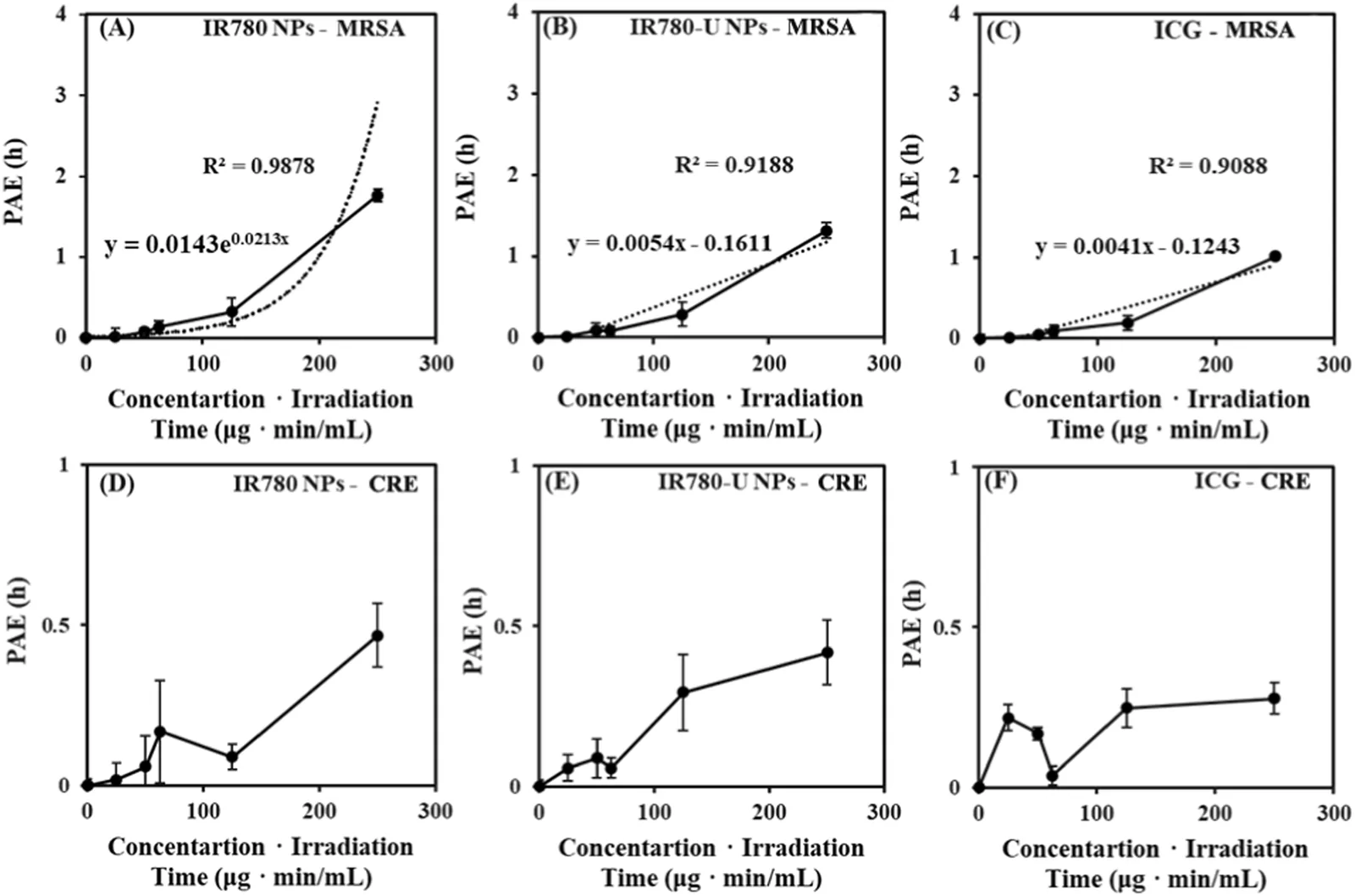
The relationship between PAE and total PTA exposure (product of PTA concentration and NIR irradiation time) for different PTAs against MRSA and the CRE isolate (n = 3). **(A)** IR780 NPs against MRSA; **(B)** IR780-U NPs against MRSA; **(C)** ICG against MRSA; **(D)** IR780 NPs against the CRE isolate; **(E)** IR780-U NPs against the CRE isolate; **(F)** ICG against the CRE isolate. (Due to the limited sample size, the 95% confidence intervals are relatively wide, and this analysis should be interpreted as a preliminary observation.)

### 3.4 The interaction mechanism between IR780 NPs and MRSA

We investigated the mechanism of the PAE-like effect of the IR780 NP. We hypothesized that the mechanism may be due to the heat released during NIR irradiation. To verify this hypothesis, we set the ambient temperature in the antibacterial test in Figure 2 to be 0 °C (under this condition, the temperature of the IR780 NP solution will be raised from 0 °C to 14.5 °C after 5 min of NIR irradiation) and repeated the experiment. The results are shown in Figure 4.

**FIGURE 4:**
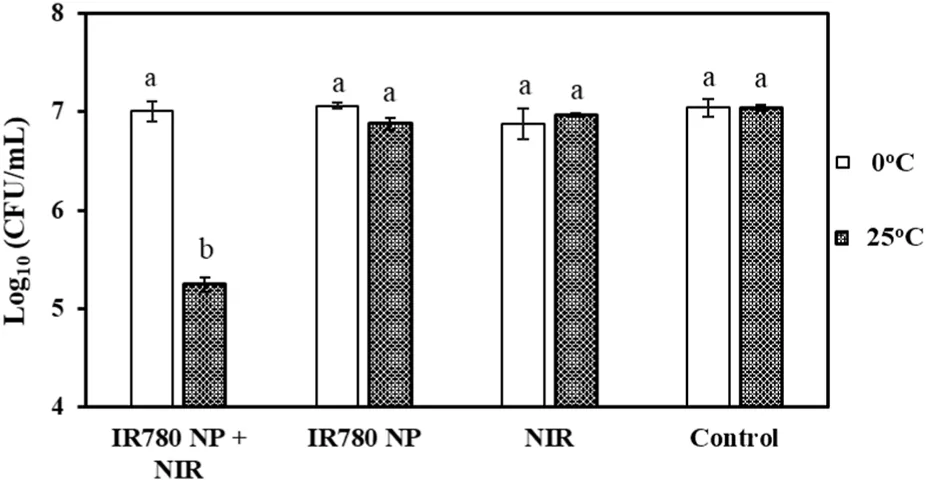
The antibacterial effect of IR780 NPs against MRSA under different ambient temperatures. No treatment was applied to the control group. The IR780 NP concentration in treated samples was 25 μg/mL, and the NIR irradiation duration was 5 min (n = 3). Different letters (a, b) above the bars indicate statistically significant differences between groups (p < 0.05, Kruskal-Wallis one-way ANOVA followed by Dunn’s *post hoc* test). Groups sharing the same letter are not significantly different (p = 0.03).

Five minutes of NIR irradiation raised the temperature of the IR780 NP solution from 0 °C to 14.5 °C. As can be seen in Figure 4, the bacterial CFU concentration in the IR780 NP + NIR group was not significantly different from the control group. The discrepancy was 0.04 log CFU/mL, while when the ambient temperature was 25 °C, the bacterial CFU concentration in the IR780 NP + NIR group decreased drastically by 1.75 log CFU/mL and was significantly different from the control group. This result hinted that thermal energy is the primary contributor to the antibacterial activity of IR780 NPs.

We then compared the cellular membrane permeability, intracellular oxidative activity, and membrane potential between IR780 NP photothermal groups and pure thermal treatment groups. As can be seen in Figure 5A, the fluorescence intensity of PI in IR780 NP (25 μg/mL) + NIR group was 1.6-fold higher than the control group, meaning that the membrane permeability was changed by the treatment. When the concentration of IR780 NP went higher, the fluorescence intensity also became higher. A similar phenomenon was observed in the thermal treatment group: 55 °C or higher thermal treatment for 10 min could significantly alter the membrane permeability of MRSA as dictated by the fluorescence intensity of PI (Figure 5B).

**FIGURE 5:**
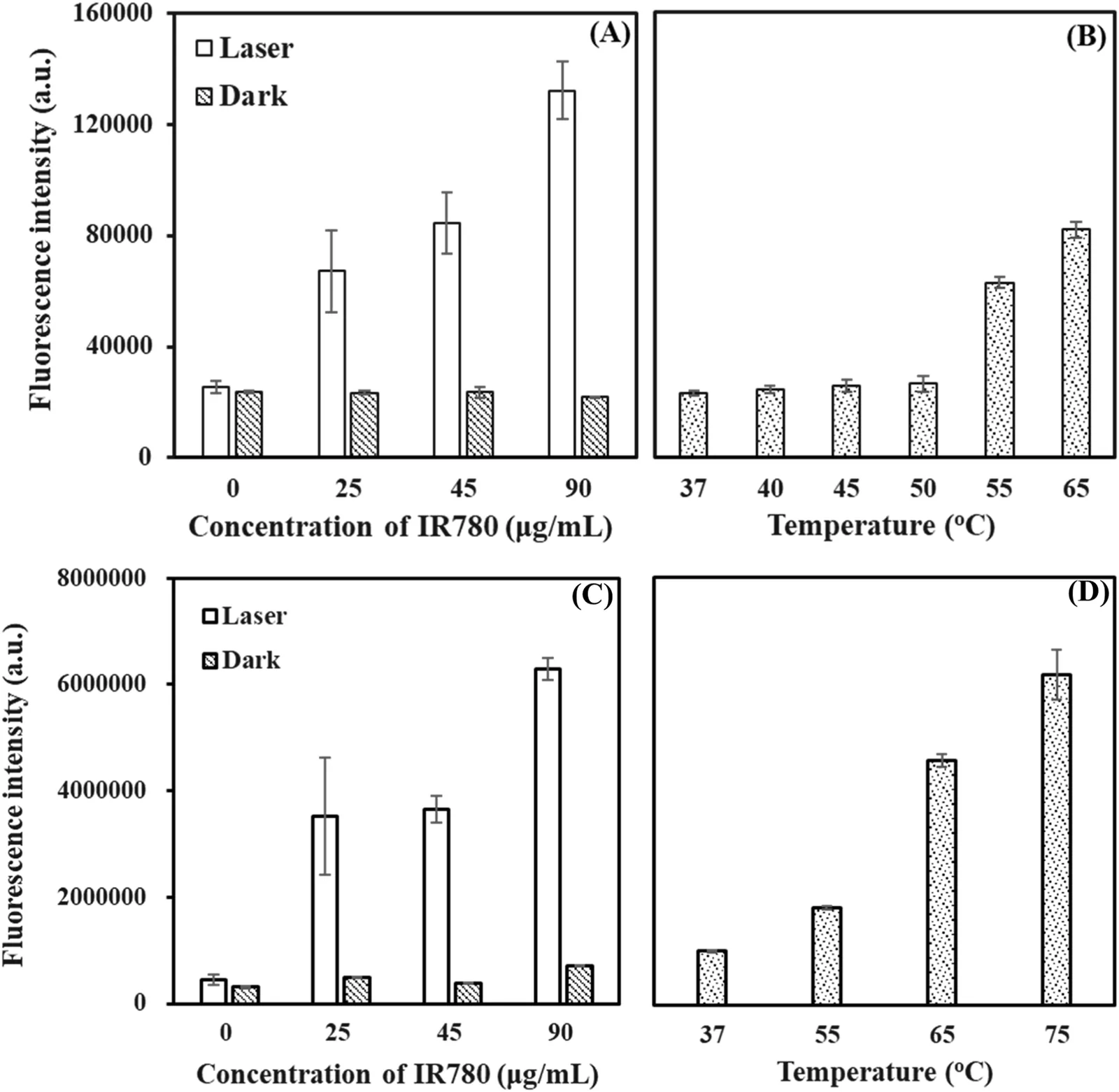
**(A,B)** Changes of MRSA membrane permeability in response to **(A)** IR780 NP photothermal treatment (NIR irradiation of 5 min), **(B)** thermal treatment (5 min) without NIR irradiation. **(C,D)** Changes of intracellular oxidative activity (indicated by DCFH-DA fluorescence) in MRSA in response to **(C)** IR780 NP photothermal treatment (NIR irradiation of 5 min), **(D)** thermal treatment (5 min) without NIR irradiation (n = 3).

The intracellular oxidative activity of MRSA increased approximately linearly with the IR780 NP concentration in PTT (Figure 5C). And 55 °C or higher thermal treatment also promoted oxidative activity intracellularly (Figure 5D). Corresponding confocal laser scanning microscope images are shown in Figure 6.

**FIGURE 6:**
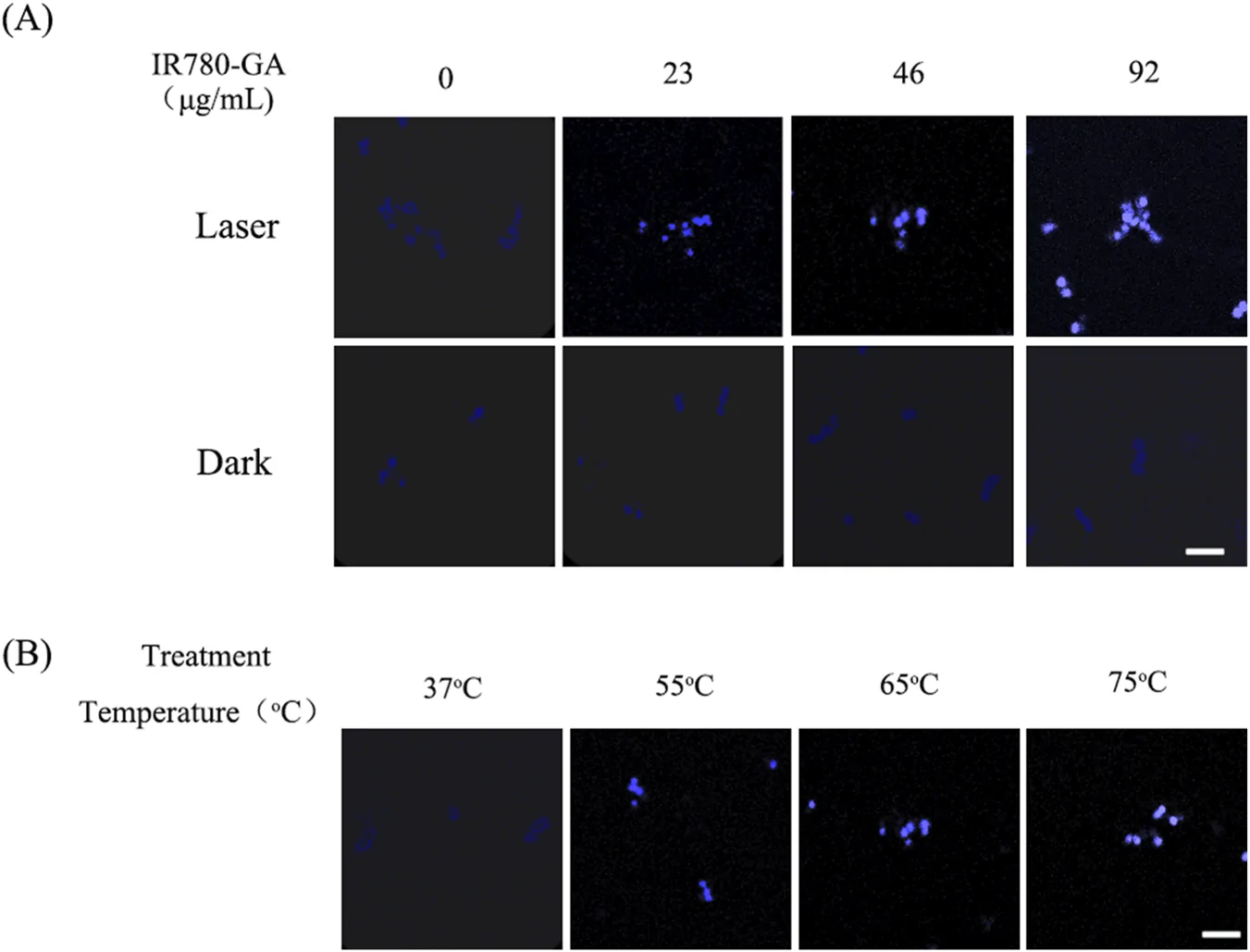
**(A)** The CLSM images of DCFH-DA-loaded MRSA after different IR780 NP photothermal treatment. The NIR treatment duration was 5 min. **(B)** The CLSM images of DCFH-DA-loaded MRSA after different thermal treatments for 5 min. Scale bar = 5 μm.

Furthermore, the fluorescence intensity of DiSC_3_(5) increased after IR780 NP photothermal treatment (Figure 7), indicating membrane depolarization of MRSA. The fluorescence intensity of DiSC_3_(5) also increased after thermal treatment (≥55 °C, 5 min). The results above indicated that IR780 NP photothermal treatment and thermal treatment shared a similar way in affecting MRSA, meaning that IR780 NP photothermal treatment probably influenced MRSA by its thermal energy.

**FIGURE 7:**
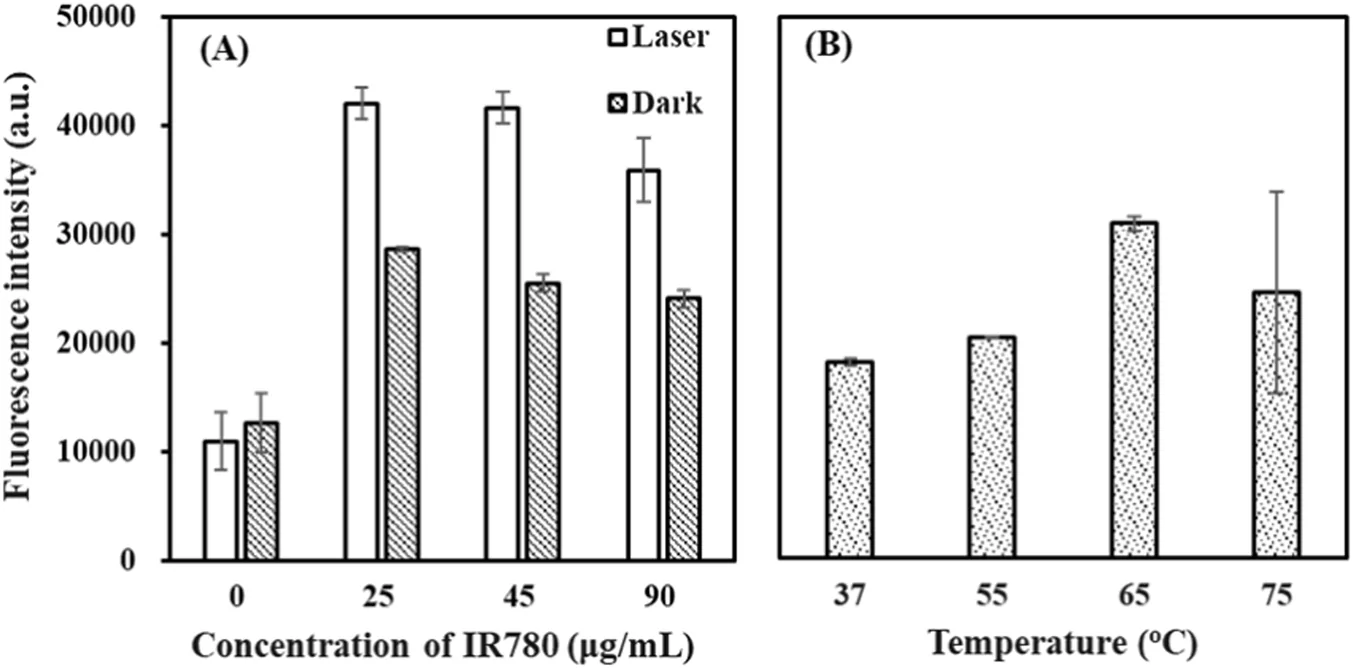
Changes of MRSA membrane potential in response to **(A)** IR780 NP photothermal treatment (NIR irradiation of 5 min), **(B)** thermal treatment (5 min) without NIR irradiation (n = 3).

## 4 Discussion

Prior work has documented different kinds of PTAs that are effective towards killing bacteria *in vivo*, including IR780, ICG, Prussian blue, etc (Li Y. et al., 2024). For example, Huang et al. developed core-shell-structured IR780@PLGA NPs that are highly efficient for antibacterial therapy (Huang et al., 2022). However, the PAE-like effect, which is highly instructive in determining treatment dosing and frequency of administration of any PTA, has never been investigated before. In this work, we chose three different kinds of PTAs: IR780 NPs with bacterial targeting ability (IR780 NP), IR780 NPs without bacterial targeting ability (IR780-U NP), and ICG, and tested their PAE-like effect against MRSA and the CRE isolate. We also explored the effect of PTA concentration and NIR irradiation duration on PAE-like effect. Our results indicated that the PAE-like effect value varied with PTA type and bacterial species under the current experimental conditions. However, given that the recognition/accumulation of IR780 NPs may differ between bacterial species, whether this variation is attributable to intrinsic differences in bacterial susceptibility or to differential PTA-bacteria interactions requires further investigation using well-defined targeting moieties (e.g., antibodies or specific peptides). PTA with targeting capability resulted in a higher PAE-like effect value, which might be due to the temperature at the bacterial surface being higher with IR780 NPs adsorbing on it. For MRSA, the PAE-like effect showed a positive correlation with total PTA exposure (the product of PTA concentration and NIR irradiation duration), with a trend roughly consistent with a nonlinear rise (R^2^ > 0.9). A similar pattern has been reported for antibiotics, where the recovery time of *E. coli* increased with the product of antibiotic exposure time and concentration (Srimani et al., 2017). However, we acknowledge that this analysis is based on a limited number of data points (n = 3 per condition), and the observed trend should be considered preliminary. Additional data points will be needed to further validate this relationship in future studies.

As for the mechanism behind the PAE-like effect, our results indicated that the antibacterial activity of IR780 NPs relied on ambient temperature. Furthermore, photothermal treatment of IR780 NPs affected MRSA in a similar way as thermal treatment: they both raised the permeability of cell membranes, promoted inner cell oxidative activity, and resulted in membrane depolarization. So, our results suggest that thermal energy is the major mechanism underlying the antibacterial and PAE-like effects of IR780 NPs, although thermally matched controls would be needed to fully exclude other contributions (Soni et al., 2020). Some bacteria may be killed instantly, and the rest might be left with nonlethal damage. This may explain the mechanism of PAE.

We acknowledge that the proposed explanation for the PAE-like effect—namely, the time required for bacteria to repair sublethal thermal damage—remains a hypothesis at this stage. Our data are consistent with this interpretation, as we observed membrane permeability changes, oxidative activity, and membrane depolarization that are known to accompany thermal injury. However, we did not directly assess repair kinetics, protein denaturation, stress-response pathways, or metabolic recovery. Therefore, the mechanistic interpretation should be viewed as a working hypothesis that requires further experimental validation in future studies.

This work provides a preliminary *in vitro* foundation for investigating treatment intervals in future *in vivo* studies.

In future work, PAE-like effect of other kinds of PTAs on different types of bacteria might be examined systematically. A better method should also be designed to understand the mechanism of their PAE.

A limitation of this study is that we did not directly quantify residual bacteria-associated PTAs after dilution. While our 1000-fold dilution protocol is standard for PAE assays (Huband et al., 2015), we acknowledge that the enhanced PAE observed in the targeted NP group could, in part, be influenced by residual PTAs. Given the intrinsic fluorescence of IR780, future studies can quantify residual PTAs by measuring the fluorescence of the bacterial pellet after dilution to address this question more definitively.

Another limitation of this study is that thermally matched controls (heat-only treatments reproducing the exact temperature-time profile of photothermal treatments) were not included. While our multiple lines of evidence point to thermal damage as the primary mechanism, we cannot entirely rule out contributions from other PTT-associated effects. Future studies incorporating such controls will be needed to definitively establish mechanistic equivalence.

Furthermore, we also acknowledge that we did not include a validated depolarization control (e.g., CCCP or valinomycin) in the DiSC_3_(5) assay, nor did we perform kinetic monitoring of fluorescence changes. While our data clearly show increased DiSC_3_(5) fluorescence following treatment—consistent with membrane depolarization—we acknowledge that inclusion of such controls would strengthen this interpretation. Future studies will incorporate these controls.

We also acknowledge that fluorescence interference controls and positive controls were not included for the mechanistic assays; these will be incorporated in future studies.

Furthermore, we acknowledge that the CRE isolate used in this study (GDB8P65M) is a single clinical isolate. Therefore, our findings regarding the PAE-like effect against CRE should be considered preliminary and should not be generalized to all carbapenem-resistant *E. coli* strains without further validation using a larger panel of diverse clinical isolates.

## 5 Conclusion

This work provides the first evidence that PAE existed in PTT. PTAs with bacterial targeting abilities had a higher PAE value. By adding bacterial targeting groups to IR780 NP, its PAE value could be raised by 34.6%. Based on our findings, we propose that the observed PAE-like effect likely results from the time required for bacteria to repair sublethal thermal damage. These insights provide a preliminary basis for designing future *in vivo* studies to optimize PTT regimens.

## Data Availability

The raw data supporting the conclusions of this article will be made available by the authors, without undue reservation.
