# Supplementary material for: The postantibiotic effect-like effect of photothermal agents: evidence from IR780 nanoparticles and indocyanine green
Source: Front Pharmacol. 2026 Sep 30;17:1939215. doi: 10.3389/fphar.2026.1939215 (PMC13661360; doi:10.3389/fphar.2026.1939215)
Supplement: Supplementary file 1 [file Image1.pdf]

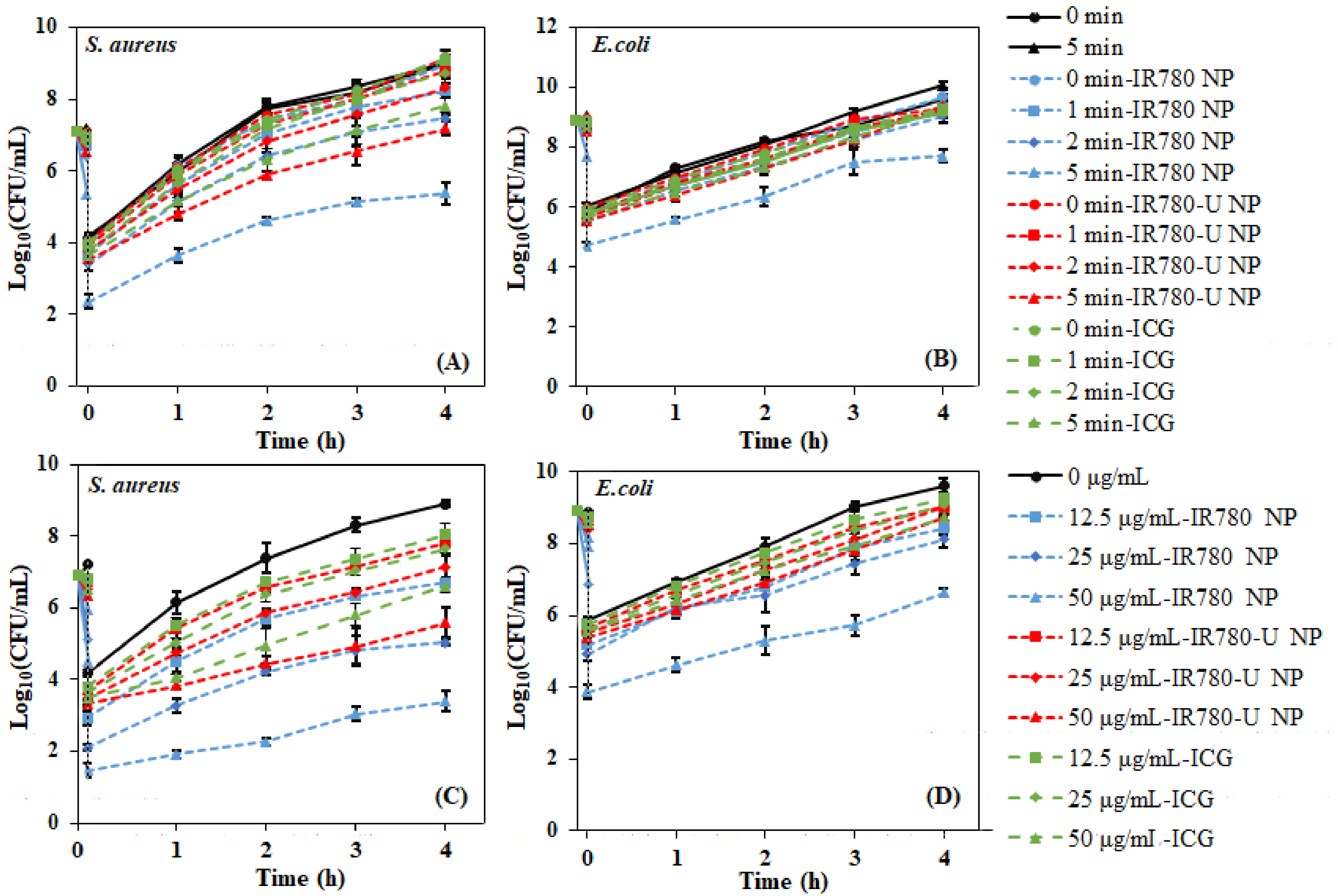

Figure S1. Regrowth curves of *S. aureus* and *E. coli* after PTA treatment under various conditions. (A) *S. aureus* treated with PTAs at 25 µg/mL with different NIR irradiation time; (B) *E. coli* treated with PTAs at 25 µg/mL with different NIR irradiation time; (C) *S. aureus* treated with different PTA concentrations with 5 min of NIR irradiation; (D) *E.coli* treated with different PTA concentrations with 5 min of NIR irradiation. (n=3)
